# Type 1 regulatory T cells specific for collagen type II as an efficient cell-based therapy in arthritis

**DOI:** 10.1186/ar4567

**Published:** 2014-05-22

**Authors:** Hélène Asnagli, Delphine Martire, Nathalie Belmonte, Julie Quentin, Hervé Bastian, Mathilde Boucard-Jourdin, Papa Babacar Fall, Anne-Laure Mausset-Bonnefont, Amélie Mantello-Moreau, Sandrine Rouquier, Irène Marchetti, Christian Jorgensen, Arnaud Foussat, Pascale Louis-Plence

**Affiliations:** 1 TxCell SA, Allée de la Nertière, Les Cardoulines, Sophia Antipolis-Valbonne, 06560 Valbonne, France; 2 Inserm, U844, University of Montpellier 1, 80 rue Augustin Fliche, 34295 Montpellier, Cedex 05, France; 3 CHU Lapeyronnie, Avenue Gaston Giraud, 34295 Montpellier, Cedex 05, France

## Abstract

**Introduction:**

Regulatory T (Treg) cells play a crucial role in preventing autoimmune diseases and are an ideal target for the development of therapies designed to suppress inflammation in an antigen-specific manner. Type 1 regulatory T (Tr1) cells are defined by their capacity to produce high levels of interleukin 10 (IL-10), which contributes to their ability to suppress pathological immune responses in several settings. The aim of this study was to evaluate the therapeutic potential of collagen type II–specific Tr1 (Col-Treg) cells in two models of rheumatoid arthritis (RA) in mice.

**Methods:**

Col-Treg clones were isolated and expanded from collagen-specific TCR transgenic mice. Their cytokine secretion profile and phenotype characterization were studied. The therapeutic potential of Col-Treg cells was evaluated after adoptive transfer in collagen-antibody– and collagen-induced arthritis models. The *in vivo* suppressive mechanism of Col-Treg clones on effector T-cell proliferation was also investigated.

**Results:**

Col-Treg clones are characterized by their specific cytokine profile (IL-10^high^IL-4^neg^IFN-γ^int^) and mediate contact-independent immune suppression. They also share with natural Tregs high expression of GITR, CD39 and granzyme B. A single infusion of Col-Treg cells reduced the incidence and clinical symptoms of arthritis in both preventive and curative settings, with a significant impact on collagen type II antibodies. Importantly, injection of antigen-specific Tr1 cells decreased the proliferation of antigen-specific effector T cells *in vivo* significantly.

**Conclusions:**

Our results demonstrate the therapeutic potential of Col-Treg cells in two models of RA, providing evidence that Col-Treg could be an efficient cell-based therapy for RA patients whose disease is refractory to current treatments.

## Introduction

Rheumatoid arthritis (RA) is a chronic autoimmune disease characterized by synovial inflammation and destruction of joint cartilage and bone and mediated by persistent synthesis of proinflammatory cytokines and matrix metalloproteinases. Proinflammatory cytokines such as interleukin 6 (IL-6), tumor necrosis factor α (TNF-α) and IL-1β are critical mediators in the inflammatory process of arthritis [1,2]. In the past several years, biologic drugs have been developed to antagonize the effector cytokines, and neutralizing TNF-α or IL-6 has been proven to be successful in the treatment of RA. Despite the clinical benefit of such biologics aimed at ensuring broad immunosuppression, a nonnegligible proportion of patients eventually escape. For example, treatment failures can be related to the development of an immune response against the biologic itself, thus leading to loss of efficacy over time [3-5]. As a consequence of these failures, there is still a need for new therapies with the aim of proactively restoring immune balance and reestablishing tolerance to joint antigens while avoiding systemic immune suppression.

Regulatory T (Treg) cells have been shown to play a crucial role in inhibiting autoimmune diseases and could be a valuable, interesting tool for use in therapeutic interventions, including in RA treatment. Indeed, Treg cells are ideal for this purpose because they suppress inflammation in an antigen-specific manner and can achieve selective and durable inhibition of pathologic inflammation without blocking protective immune responses against infection. The results of many animal model studies [6-10], as well as clinical studies, have indicated a link between the efficacy of therapies against arthritis and the increase in the number or function of Treg cell populations [11-14]. In addition, oral tolerization protocols developed several years ago have shown disease reduction in RA murine models and have recently been associated with the development of a population of Treg cells that suppress inflammation via IL-10 production [15,16]. More importantly, treatment of RA patients with anti-TNF antibodies has been shown to induce differentiation of a potent population of Treg cells with suppressive activity that is dependent upon transforming growth factor β (TGF-β) and IL-10 [12,13].

Because of the heterogeneity of human Treg cells, there is no consensus to date about which Treg cell population is optimally suitable for clinical use. Investigators in several phase I clinical trials have tested the ability of *ex vivo*–expanded CD4^+^CD25^+^ Treg cells to prevent graft versus host disease after allogeneic bone marrow transplantation [17-19]. The use of such Treg cell populations has been hampered by the low number of circulating Tregs in humans and by the need to expand large amounts of suppressive cells for clinical use. Based on the therapeutic potential of type 1 regulatory T (Tr1) cells in inflammatory bowel disease (IBD) models [20] and on the possibility of generating large numbers of antigen-specific Tr1 cells *in vitro*[21,22], a phase I/II clinical trial in which the researchers used ovalbumin-specific type 1 regulatory T (ova-Treg) cells has been performed in severe Crohn’s disease patients with production of very promising clinical data and indications of safety and efficacy [23]. These results and the possibility of isolating and expanding functional collagen type II–specific type 1 regulatory T (Col-Treg) cell clones from RA patients [22], suggesting that Tr1 cell–based therapy could be a novel therapeutic strategy for treating patients with RA.

Tr1 cells are characterized by the secretion of high amounts of IL-10. Adoptive transfer of antigen-specific Tr1 cells has been shown to be efficacious in suppressing inflammation in a variety of animal models for chronic inflammatory and autoimmune diseases, such as colitis, type 1 diabetes, multiple sclerosis, allergy and transplantation [20,24-28]. In these settings, the putative mechanism of action of antigen-specific Tr1 cells can be summarized in three steps: (1) homing to the site of inflammation, (2) local recognition of the specific antigen presented by local antigen-presenting cells and (3) local suppression of inflammation mediated by soluble factors and immunomodulatory surface molecules. By their mode of action, Tr1 cells are able to deliver local immunosuppression to the inflamed tissue.

Our goal in the present study was to evaluate the therapeutic potential of Col-Treg cells in experimental models of arthritis in order to propose their use in a new cell-based immunotherapy in joint inflammatory diseases, especially for patients with RA refractory to available treatments.

## Methods

### Ethical approval

All experiments were performed in accordance with national guidelines and approved by the local ethics committee (Committee on the Ethics of Animal Experiments in Languedoc-Roussillon (CEEA-LR-1067)) and the French National Authority for Health (A06-152-10 and B34-172-36).

### Mice

BALB/c and DBA/1 mice were obtained from Harlan Laboratories (Indianapolis, IN, USA). DO11.10 and CD90.2 congenic BALB/c mice were kindly provided by H Yssel and J Hernandez, respectively, and mice were bred in our own animal facility. Transgenic mice carrying the rearranged Vα11.1 and Vβ 8.2 T-cell receptor (TCR) chain genes isolated from a collagen type II (Col II)–specific T-cell hybridoma were kindly provided by REM Toes (Leiden University Medical Center, Leiden, the Netherlands) with approval from W Ladiges [29].

### Generation of collagen-specific and ovalbumin-specific type 1 regulatory T cells

Col-Treg clones were generated from Col II–specific transgenic mice. Briefly, splenocytes were stimulated in the presence of bovine Col II (0.5 μg/ml) (MD Biosciences, Zurich, Switzerland), recombinant human (rhu) IL-10 (50 ng/ml) (R&D Systems, Minneapolis, MN, USA) and anti-IL-4 antibodies (10 μg/ml) (clone 11B11; eBioscience, San Diego, CA, USA). Similarly, ova-Treg clones were generated as previously described [26] from DO11.10 transgenic mice, stimulated in the presence of 0.3 μM OVA_323–339_ peptide (Bachem, Bubendorf, Switzerland). In both cases, recombinant murine IL-10 (5 ng/ml; R&D Systems) was added on day 2, and cells were cloned by limiting dilution in the presence of coated anti-CD3 monoclonal antibodies (mAbs) (5 μg/ml) (clone 145.2C11; BD Biosciences, San Jose, CA, USA) in the presence of irradiated syngeneic splenocytes. T-cell clones were then expanded in the presence of rhu IL-2 (aldesleukin; Novartis, Basel, Switzerland) with coated anti-CD3 (5 μg/ml) and soluble anti-CD28 (1 μg/ml) mAbs. T cells were further expanded in the presence of anti-CD3/anti-CD28 mAb–coated beads (Invitrogen, Carlsbad, CA, SUA) with addition of IL-2 on day 2, with an average 240-fold expansion over the course of 2 weeks.

### Characterization of collagen-specific regulatory T cells

Cytokines in the culture supernatants were quantified by enzyme-linked immunosorbent assay (ELISA) with IL-4 and IL-10 (BD Biosciences) and interferon γ (IFN-γ) (R&D Systems) following 48 hours of polyclonal stimulation (coated anti-CD3 and soluble anti-CD28 mAbs) of Treg cells in expansion (no IL-10 added). Intracellular cytokine secretion staining of CD4^+^Vβ8.2^+^ clones was analyzed by fluorescence-activated cell sorting (FACS) using fluorescent dye–conjugated anti-IL-10, anti-IFN-γ, anti-IL-4, anti-IL-13 and anti-IL-17 mAbs (eBiosciences or BD Biosciences) after 4 hours of stimulation with phorbol 12-myristate 13-acetate (50 ng/ml) and ionomycin (1 μg/ml) in the presence of brefeldin A (10 μg/ml).

Selected Col-Treg clones were further characterized by FACS analysis using fluorescent dye–conjugated mAbs specific for CD25, FoxP3, CD127, CD62L (BD Biosciences) and glucocorticoid-induced tumor necrosis factor family receptor (GITR), cytotoxic T lymphocyte-associated antigen 4 (CTLA-4), granzyme B, CD39 and CD73 (eBiosciences). Cells were stained according to standard procedures. Data were acquired on a FACSAria flow cytometer (BD Biosciences), and the results were analyzed with FACSDiva software (BD Biosciences).

The immunosuppressive function of Col-Treg clones was evaluated by cell contact–independent *in vitro* assay in transwell plates using a method adapted from that described by Battaglia *et al*. [30]. Briefly, we used as responder cells freshly isolated splenocytes stained with carboxyfluorescein diacetate succinimidyl ester (CFSE) (Invitrogen) and plated them in the presence of anti-CD3 mAbs (5 μg/ml) in the bottom well of a 96-transwell plate (0.8 × 10^6^ cells/well). We added either medium alone or selected Col-Treg clones (0.05 × 10^6^ cells/well) activated with anti-CD3/anti-CD28-coated beads in the upper well. After 3 days, the proliferation of responder cells was evaluated by FACS analysis of CFSE dilution staining on CD4^+^ cells. The suppressive capacity of clones was measured by comparing the percentage of CFSE^+^ divided cells obtained in the presence of Col-Treg clones with the percentage of CFSE^+^ divided cells obtained without any added cells.

### Induction and clinical evaluation of collagen antibody–induced arthritis

We delivered a cocktail consisting of 5 or 6 mg of arthritogenic antibodies specific for Col II epitopes (MD Biosciences) by intraperitoneal injection into male 9-week-old DBA/1 mice. On day 3 after the cocktail injection, we intraperitoneally injected into the mice 100 μg of lipopolysaccharide (LPS) (MD Biosciences), and, a few hours later, we injected Col-Treg cells by intravenous retro-orbital delivery into the venous sinus. Starting from day 3 after the initial injection, disease severity was scored daily based on clinical scores according to the following scale: 0 = normal, 1 = weak swelling, 2 = significant swelling associated with redness, 3 = intermediate swelling associated, or not, with redness and 4 = maximal swelling and/or redness in all inflamed digits. A total score of 20 was recorded for each mouse and added to a total score of 2 based on the number of inflamed digits [31]: 0 = no inflamed digits, 0.5 = zero to five inflamed digits, 1 = six to ten inflamed digits, 1.5 = one to fifteen inflamed digits and 2 = 16 or more inflamed digits. In addition to the clinical score, the mice were analyzed for body weight loss.

### Collagen-induced arthritis induction and evaluation

We immunized male 9- to 12-week-old DBA/1 mice at the base of the tail with 100 μg of bovine Col II (MD Biosciences) emulsified in complete Freund’s adjuvant. On day 21 after arthritis induction, we delivered a booster immunization at the base of the tail with 100 μg of bovine Col II emulsified in incomplete Freund’s adjuvant (IFA). We injected Col-Treg cells (intravenous retro-orbital injection) into the mice on day 20, 22 or 28 after arthritis induction. From day 21 onward, the thickness of each hind paw was measured with a caliper three times per week, and the severity of arthritis was graded using the same clinical scale described for collagen antibody–induced arthritis (CAIA). The score data are expressed as means ± SEM on a given day.

### Histopathology

At experimental endpoints, joints were collected, fixed in 10% neutral buffered formalin, decalcified in 5% ethylenediaminetetraacetic acid–based decalcification solution for several days and embedded in paraffin, and sections were stained with hematoxylin and eosin. Each joint (ankle and wrist) was scored in a blinded manner by two independent histopathologists (MD Biosciences). Infiltration was scored as follows: 0 = no change, 1 = modest leukocyte infiltration into synovial tissue with no fluid leukocytes, 2 = modest leukocyte infiltration into synovial tissue in fluid phase with no loss of synovial architecture and 3 = gross leukocyte infiltration into synovial membrane and fluid space with significant loss of synovial and articular architecture. Erosion was scored as follows: 0 = no abnormality, 1 = fibrillation of cartilage and/or mild erosive infiltration of periosteal and subchondral bone, 2 = moderate fibrillation and loss of cartilage and/or erosive infiltration of periosteal and subchondral bone and 3 = significant loss of cartilage and/or erosive infiltration of periosteal and subchondral bone.

### Antibody responses

Serum levels of immunoglobulin G1 (IgG1), IgG2a and total IgG antibodies against bovine Col II were measured by standard ELISA using pooled sera from arthritic mice as a reference standard and assigned an arbitrary level of antigen-specific antibodies [32].

### Detection of collagen-specific regulatory T cells by quantitative PCR

For the detection of Col-Treg cells by PCR, primers and dual-label probe (6-carboxyfluorescein and Toll/IL-1 receptor domain–containing adapter inducing IFN-β, or FAM-TRAM; Sigma-Aldrich, St Louis, MO, USA) were designed for the specific TCR VDJ (variable, diversity, joining) recombination genetic sequence expressed by transgenic mice [29]. Quantitative PCRs were performed on total tissue-derived genomic DNA extracted using the GenElute Mammalian Genomic DNA Miniprep Kit (Sigma-Aldrich). The data derived from real-time quantitative PCR (DNA Engine Opticon 2 System; Bio-Rad Laboratories, Hercules, CA, USA) were expressed as the number of transgenic positive cells (determined based on standard curves using dilution of transgenic cells in nontransgenic cells) per miligrams of tissue or per organ, for lymph nodes (LNs) and eyes.

### Ovalbumin-specific delayed-type hypersensitivity

We injected CFSE-labeled, ova-specific CD4 cells (3 to 4 × 10^6^ cells isolated from DO11.10 mice) into male 9-week-old BALB/c mice. On the day afterward (day 0), these mice were immunized by subcutaneous injection at the tail base with ova (100 μg) emulsified in IFA. On day 5, injection of ovalbumin/phosphate-buffered saline (ova-PBS) (30 μg) was performed in one hind paw, and PBS was injected into the other contralateral paw. On the same day, 1 × 10^6^ ova-specific Treg cells were injected intravenously and the mice were killed 48 hours later. Draining lymph nodes (DLNs) were recovered and counted, and 2 × 10^6^ cells were stained with CD4 and KJ1.26 mAbs to determine the percentage of KJ1.26^+^ effector cells in the DLNs and the dilution of CFSE staining. For the homing experiment, the same procedures were carried out using CD90.1 BALB/c congenic mice and injecting a higher dose of ova-Treg cells (10 × 10^6^) were injected. The CD90.2 congenic marker was used to track the injected Treg cells by FACS.

### Statistical methods

Statistical analyses were performed by nonparametric Mann–Whitney *U* test with InStat software (GraphPad Software, La Jolla, CA, USA). A *P*-value <0.05 was considered statistically significant.

## Results

### Collagen II–specific type 1 regulatory T cell clone characterization

**Figure 1:**
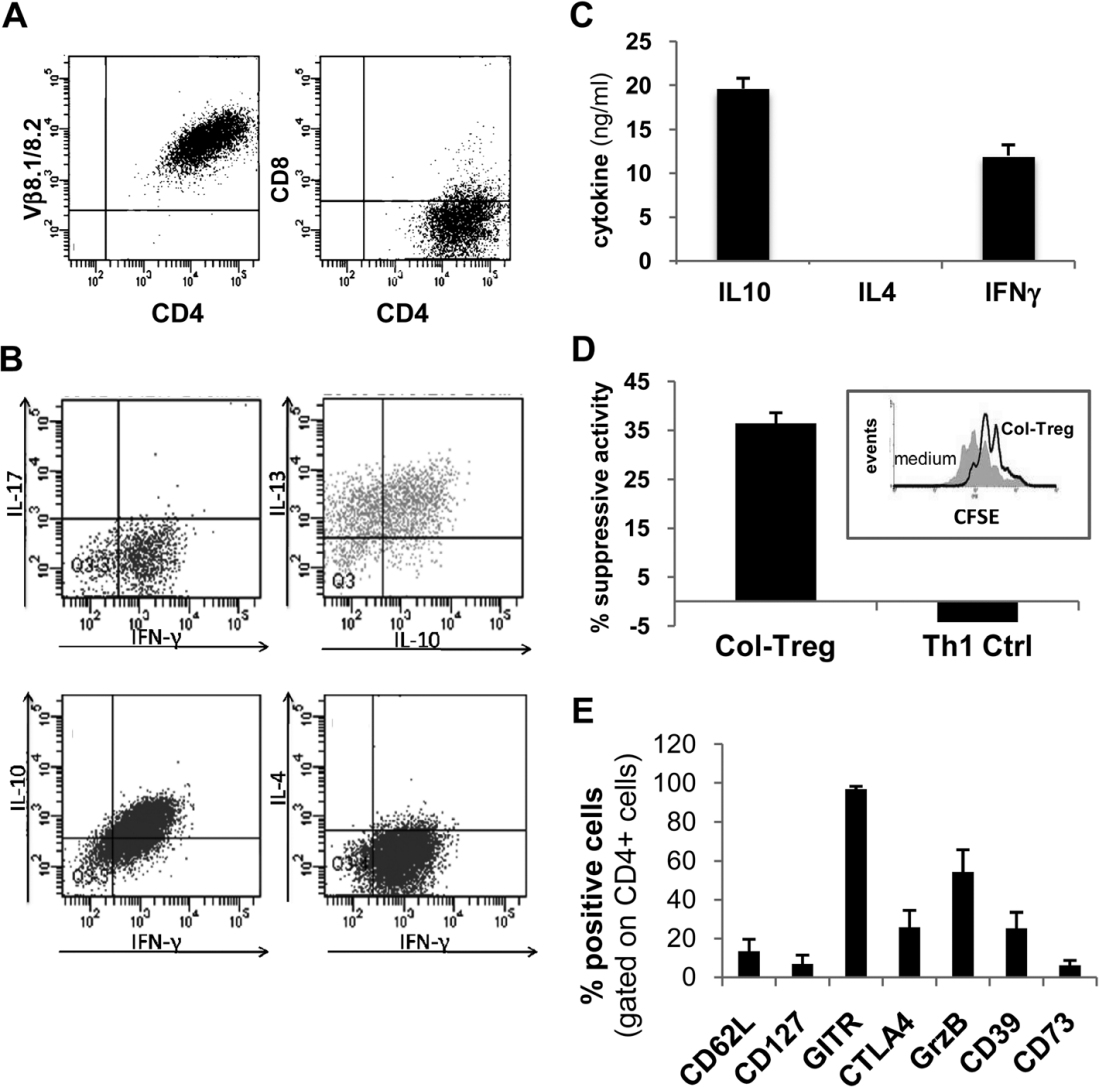
**Phenotypic characterization of the collagen type II–specific type 1 regulatory T cell clones. (A)** Graphed data of representative fluorescence-activated cell-sorting (FACS) analysis of the selected clones for the expression of T-cell receptor Vβ8 and CD4. **(B)** Graph illustrating the results of representative FACS analysis of intracellular cytokine staining of collagen type II–specific type 1 regulatory T cell (Col-Treg) clones following 4 hours of polyclonal stimulation. IFN, Interferon; IL, Interleukin. **(C)** Graph showing the cytokine secretion profile of three representative Col-Treg clones. The cytokine levels were quantified by enzyme-linked immunosorbent assay in the culture supernatants after 48 hours of polyclonal stimulation. The data are expressed as mean ± SEM. **(D)** Graph describing the immunosuppressive activity of Col-Treg clones, measured in a cell contact–independent assay by carboxyfluorescein diacetate succinimidyl ester (CFSE) dilution, after 3 days of coculture with freshly isolated splenocytes stimulated with anti-CD3 antibody. The data are representative of at least seven clones. Th1, Type 1 T helper cell. **(E)** Graph of the expression levels of several phenotypic markers following 24 hours of polyclonal stimulation. Values are mean ± SEM of the percentage of positive cells for each marker. The data are representative of three to eighteen clones.

Col-Treg clones were expanded *in vitro* from Col II–specific TCR transgenic mice in the presence of IL-10 as previously described for antigen-specific Tr1 clones in both mice and humans [20,21,26]. After expansion, clones were selected based on Col II–specific TCR Vβ8 and CD4 expression (Figure 1A) as well as on their cytokine secretion profile: IL-10^high^IL-4^neg^IFN-γ^int^ (Figure 1B and C). Additional characterization showed that selected Col-Tregs coproduce IL-13 together with IL-10, but do not express IL-17 (Figure 1B), as recently described for human ova-Treg cells [23]. The selected Col-Treg clones were further characterized based on their immunosuppressive activity in a cell-contact–independent *in vitro* assay. In contrast to control type 1 T helper (Th1) cells, Col-Treg clones were able to significantly inhibit proliferation of anti-CD3 activated CD4^+^ T cells (Figure 1D). Quantitation of their suppressive capacity showed 30% to 40% inhibition of the proliferation of CD4^+^ effector T cells (Figure 1D) concomitantly with reduction of IFN-γ levels produced by CD4^+^ T cells (data not shown).

As previously shown for Tr1 cells [21,22,28,33], and in contrast to thymus-derived CD4^+^CD25^+^ Tregs, Col-Treg clones do not constitutively express high levels of FoxP3 and CD25 (data not shown). In addition, upon activation, Col-Treg cells display a phenotype of induced Treg (iTreg) cells with low expression levels of CD127 and CD62L and induction of several activation markers, including GITR, granzyme B, CTLA-4 and CD39 (Figure 1F). The expression of such markers has been described previously for natural Treg (nTreg) cells and iTreg cells [34-36], as well as for Tr1 cells [21,28].

### Collagen type II–specific type 1 regulatory T cell clones inhibit acute arthritis

**Figure 2:**
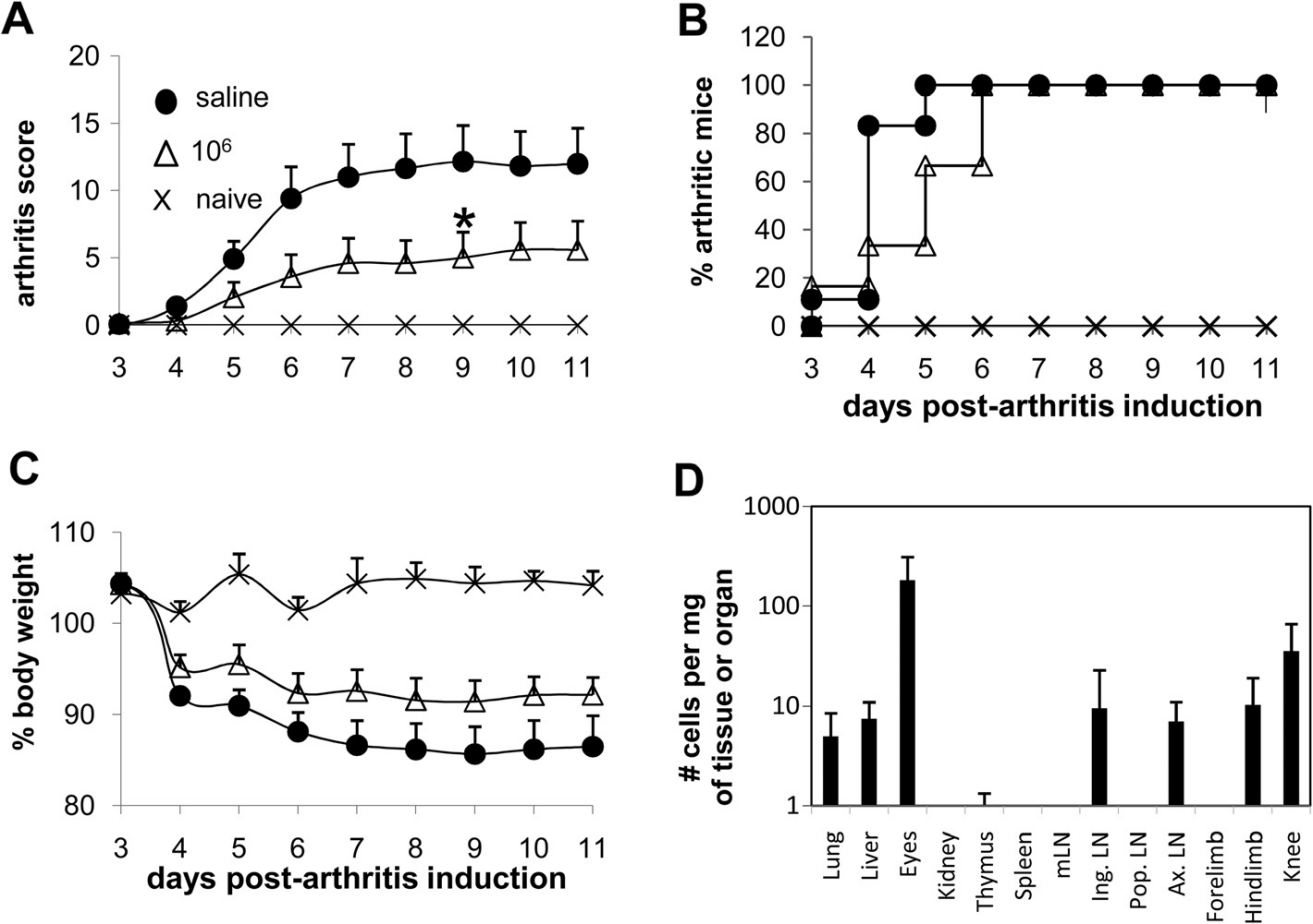
**Single administration of collagen type II–specific type 1 regulatory T cell cells reduces acute collagen-antibody-induced arthritis.** We administered intraperitoneal injections of lipopolysaccharide (100 μg) into DBA/1 mice at day 3 after arthritis induction, followed 2 to 4 hours later by intravenous injections of 1 × 10^6^ collagen type II–specific type 1 regulatory T cell (Col-Treg) clones (open triangles; *n* = 6) or saline buffer solution (closed circles; *n* = 6). Mice not injected with antibody were used as controls (X’s; *n* = 2). After T-cell infusion, the mice were evaluated daily for clinical signs of arthritis. **(A)** Graphed mean ± SEM data of the severity scores of arthritic mice are shown. Differences were analyzed by nonparametric Mann–Whitney *U* test. The data are representative of two independent experiments. *P < 0.05 with 95% confidence interval for comparison of arthritic and saline-injected control mice. **(B)** Disease incidence represents the percentage of arthritic mice at each time point. **(C)** Percentages of body weight loss are shown. **(D)** Graph illustrating the trafficking of type 1 regulatory T cell clones analyzed 24 hours after intravenous infusion of 1 × 10^6^ cells in collagen antibody–induced arthritis mice (*n* = 6). The data are expressed as mean ± SEM of the number of transgenic positive cells detected in various organs, including joint tissues, determined by quantitative PCR using standard curves. LN, Lymph node. mLN, Mesentheric LN. Ing. LN, Inguinal LN. Pop. LN, Popliteal LN. Ax. LN, Axillary LN.

To evaluate the therapeutic potential of Col-Treg cells in arthritis, Col-Treg clones were first tested in a model of model acute arthritis induced by the administration of arthritogenic antibodies (CAIA) (reviewed in [37]). Col-Tregs (1 × 10^6^ cells per animal at day 3) were injected once before the administration of LPS and disease onset. In this semipreventative treatment regimen, Col-Treg cells were able to significantly reduce paw swelling, arthritis severity and body weight loss (Figure 2). Although all mice treated with Col-Treg cells developed arthritis by the end of the experiment, we observed a slight but significant delay, compared with control mice, of paw swelling in association with disease progression (Figure 2B). Migration of the injected Col-Treg cells was evaluated in this model of acute arthritis. Twenty-four hours after the injection, cells were distributed in highly vascularized tissues, such as lung and liver, as well as in several peripheral DLNs and joints. As expected after intravenous retro-orbital sinus injection, a high number of cells were still present in the eyes (Figure 2D).

### Collagen type II–specific type 1 regulatory T cell clones inhibit chronic arthritis

As RA develops as a chronic disease, we tested Col-Treg clones in the CIA model, which mimics the human pathology involving both T- and B-cell responses [38]. Col-Treg clones were first injected as a semipreventative treatment either before (day 20) or after (day 22) the recall of the immunization. In these experiments, Col-Treg clones (1 × 10^6^ cells per animal) reduced disease severity as shown by a decrease in arthritis score and prevented body weight loss. In addition, 3 weeks after treatment, though 100% of the animals in the control group developed arthritis, only 60% to 70% of the mice in the Treg-treated group developed the disease (data not shown).

**Figure 3:**
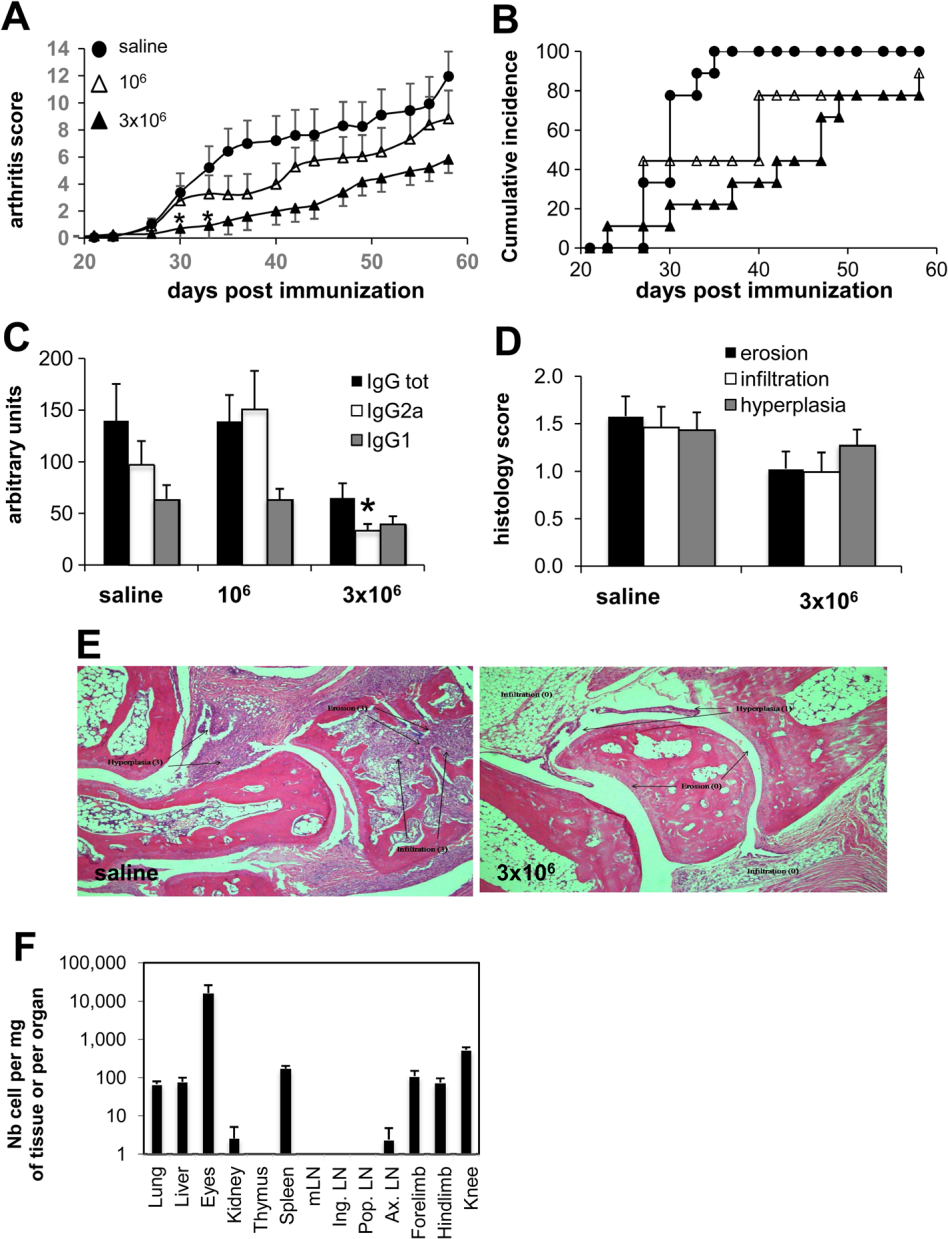
**Decrease of inflammation, bone erosion and B-cell response following injection of collagen type II–specific type 1 regulatory T cells at day 20 in murine collagen-induced arthritis.** In the DBA/1 mice, we administered intravenous injections of collagen type II–specific type 1 regulatory T cell (Col-Treg) clones in quantities of 1 × 10^6^ (open triangles; *n* = 9) or 3 × 10^6^ (closed triangles; *n* = 9) or saline buffer solution (closed circles; *n* = 9) at day 20 postimmunization. After the T-cell infusions, the mice were clinically monitored every other day. **(A)** Graphed mean ± SEM data of the arthritis severity scores are shown. Differences were analyzed by nonparametric Mann–Whitney *U* test (*P < 0.05 with 95% confidence interval represent statistically significant differences between the arthritic and saline-injected mice). **(B)** The disease incidence in each group of mice is shown. **(C)** Bovine collagen II–specific total immunoglobulin G (IgG), IgG1 and IgG2a levels in sera collected at the time mice were killed. Values expressed in arbitrary units are mean ± SEM. Asterisk represents a significant difference between arthritic and saline-injected mice. **(D)** At the time mice were killed, tissue sections were taken from the hind paws and forepaws for histological staining and scored for erosion, infiltration and hyperplasia. The values shown are mean ± SEM of each paw from nine mice per group. **(E)** Representative images of histological sections of hind paws stained with hematoxylin and eosin in control mice (left panel) or Col-Treg–treated mice (right panel). Arrows indicate hyperplasia, inflammation or erosion. Original magnification of both images = 40×. **(F)** Trafficking of type 1 Treg clones was analyzed 24 hours after intravenous retro-orbital infusion of 3 × 10^6^ Treg cells in various organs, including joint tissues (*n* = 9). LN, Lymph node. Nb, Number. mLN, Mesentheric LN. Ing. LN, Inguinal LN. Pop. LN, Popliteal LN. Ax. LN, Axillary LN.

To assess whether we could improve the clinical benefit of treatment by increasing the number of Col-Treg cells, we injected either 1 × 10^6^ or 3 × 10^6^ Col-Treg cells into the mice on day 20. The severity and incidence of arthritis (Figures 3A and 3B), as well as body weight loss (data not shown), were significantly reduced in the Treg-treated group. Interestingly, a significant reduction of Col II–specific antibody titers, in particular a reduction of the Th1-associated IgG2a isotype antibody level, was observed following injection of 3 × 10^6^ Col-Tregs (Figure 3C). We observed that the clinical response was associated with a reduction in histology scores, particularly a significant decrease in erosion, at a late stage (Figure 3D and 3E). This small difference could be explained by the fact that at the time the mice were killed, 80% of Treg-treated mice had arthritis. We also monitored the distribution of Col-Treg clones in the CIA model. As we observed in the CAIA acute arthritis model, Col-Treg cells in CIA mice migrated in highly vascularized organs, such as lung, liver and kidneys, and within joints and several LNs (Figure 3F). In addition, within 24 hours postinfusion, Col-Treg cells were detected in the spleens of CIA mice, whereas they were undetectable in CAIA mice. We performed similar analyses at 2 and 4 months after Treg injection and found that only one of seventeen mice showed a positive signal in one axillary LN, suggesting absence of uncontrolled proliferation of Col-Treg cells, even in the presence of both antigens and inflammation (data not shown).

### Clinical benefit of collagen type II–specific type 1 regulatory T cell treatment at disease onset

**Figure 4:**
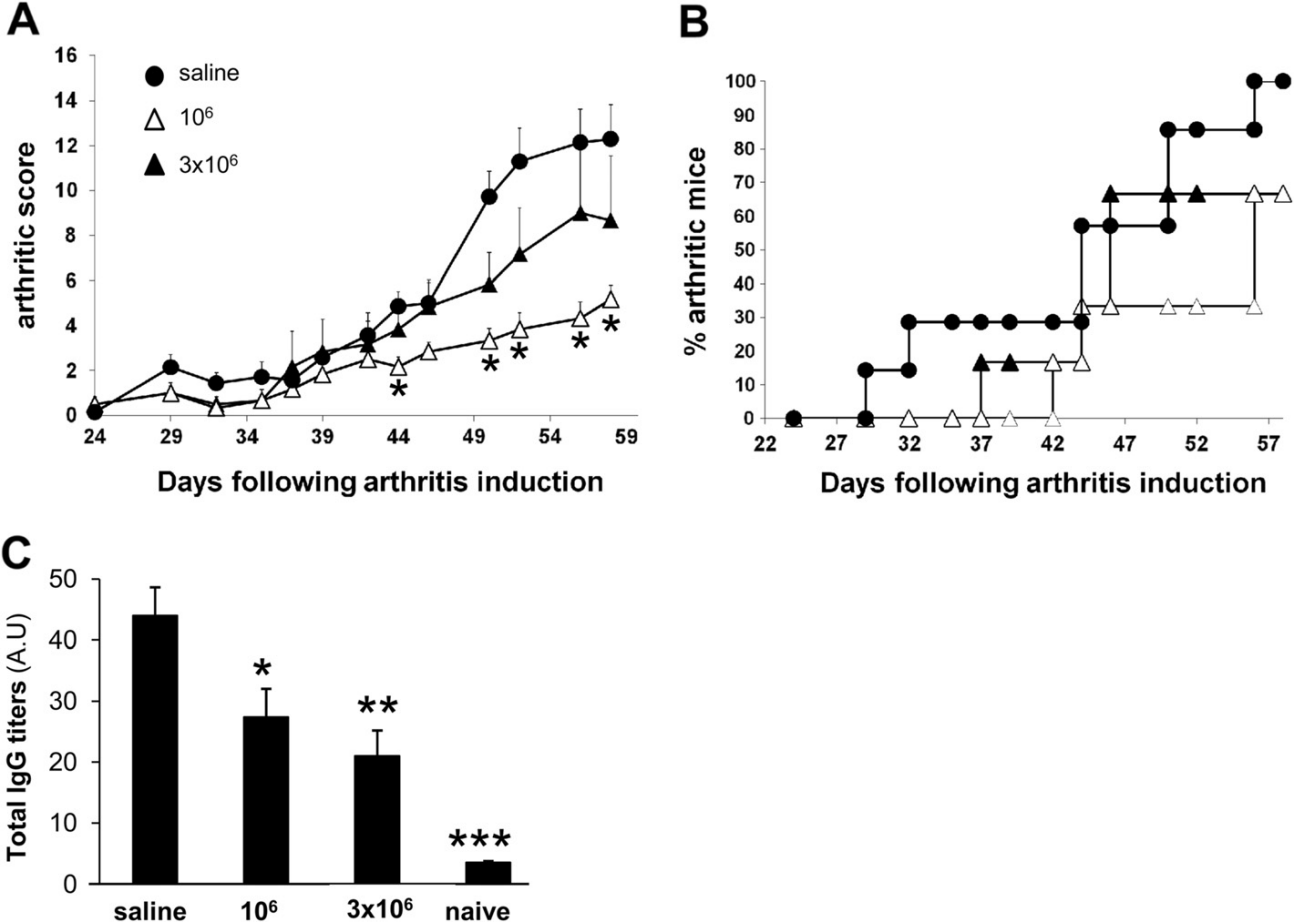
**Collagen type II–specific type 1 regulatory T cells reduce the severity of ongoing collagen-induced arthritis.** At day 28 after induction of arthritis, bovine collagen II–immunized DBA/1 mice received injections of either 1 or 3 × 10^6^ collagen type II–specific type 1 regulatory T (Col-Treg) cells, and clinical and biological monitoring of arthritis were continued until day 56. **(A)** Graphed mean ± SEM data of arthritis severity scores from day 24 of saline-injected mice (closed circles; *n* = 7) or mice injected with 3 × 10^6^ Col-Treg cells (closed triangles; *n* = 6) or 1 × 10^6^ Col-Treg cells (open triangles; *n* = 6). *P < 0.05 (Mann–Whitney *U* test) indicates statistically significant difference in severity scores between mice injected with Col-Treg cells vs. saline buffer. **(B)** Graph describing disease incidence in each group of mice. **(C)** Bovine collagen II–specific total immunoglobulin G (IgG) levels in sera collected at the time the mice were killed. Values are expressed in arbitrary units (A.U) and represent the mean ± SEM. Similar results were obtained in three independent experiments. Asterisks represent statistically significant differences in IgG levels between arthritic mice and mice injected with saline buffer. Differences were analyzed by Mann–Whitney *U* test (*P < 0.05, **P < 0.005, ***P < 0.0005 with 95% confidence intervals).

In order to further assess their therapeutic potential, we injected Col-Treg cells later in the disease course, at day 28, early in disease onset. As observed for semipreventative treatment (Figure 3), both 3 × 10^6^ and 1 × 10^6^ Col-Treg cell doses delayed arthritis onset and decreased its incidence (Figure 4B). More importantly, we observed a significant decrease in arthritis severity from day 44 following injection of 1 × 10^6^ cells (Figure 4A). The therapeutic effect was associated with a significant decrease in anti-Col II–IgG antibodies for both tested doses (Figure 4C). No preferential decrease in the Th1-associated IgG2a isotype was observed following injection at day 28, in contrast to the results observed after injection at day 20 (Figure 3), suggesting that an injection of Treg cells at the onset of clinical scoring did not impact the Th1–Th2 cell balance. Altogether, these results underscore the therapeutic potential of Col-Treg cells for clinical application and the need to clearly define the optimal dose of cells and the optimal timing of Treg cell–based therapy in a defined experimental model.

### Antigen-specific type 1 regulatory T cells migrate to draining lymph nodes and dampen proliferation of effector T cells

**Figure 5:**
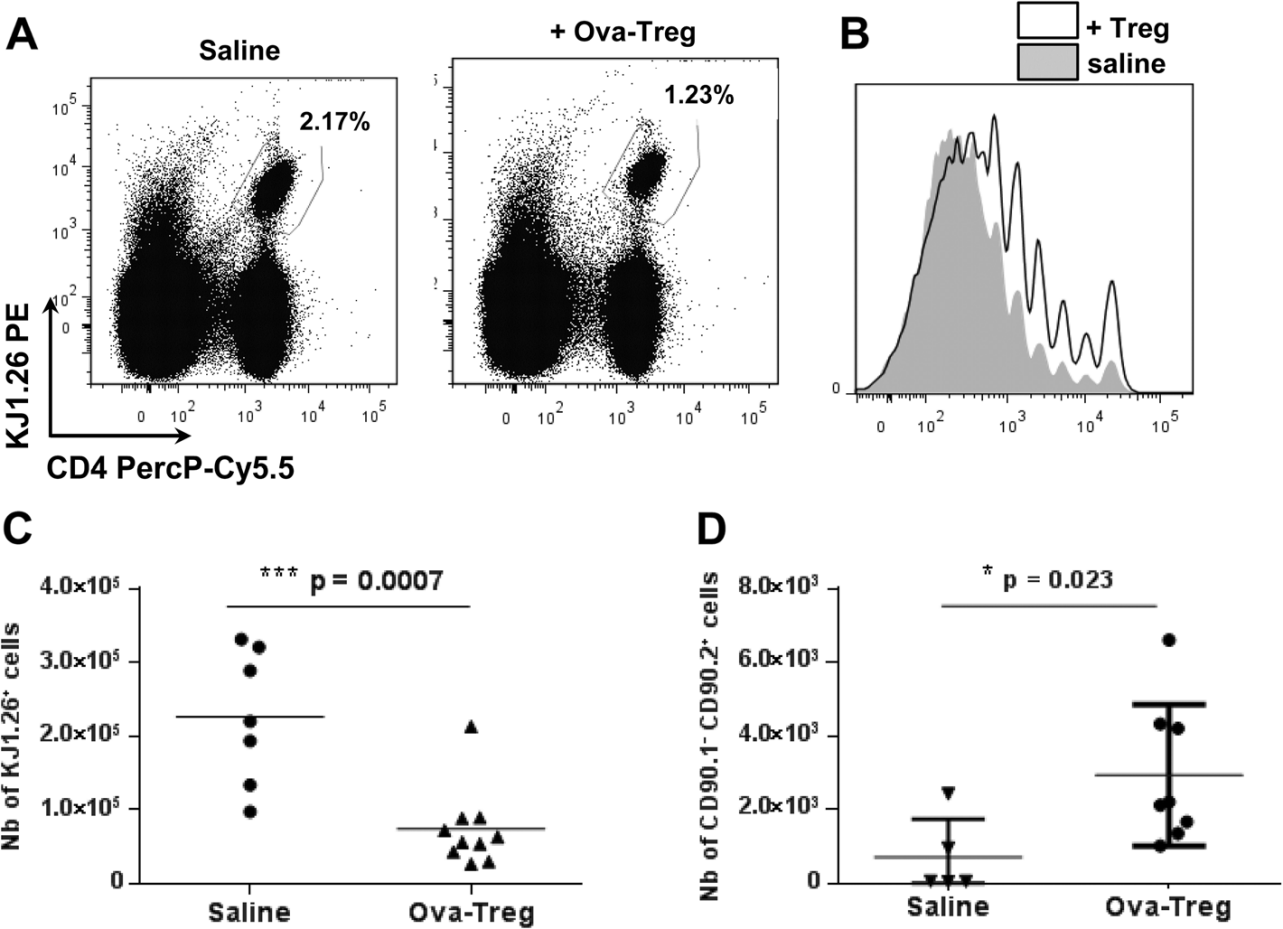
**Antigen-specific type 1 regulatory T cells dampen the proliferation of effector T cells.** BALB/c mice received injections of carboxyfluorescein diacetate succinimidyl ester (CFSE)–labeled, ovalbumin (ova)-specific effector CD4^+^ cells on the day before subcutaneous immunization with a mixture of ovalbumin and incomplete Freund’s adjuvant. At day 5, either phosphate-buffered saline (PBS) (*n* = 7) or 1 × 10^6^ ova-specific type 1 regulatory T (ova-Treg) cells (*n* = 10) were injected intravenously. The mice received ova injections into their hind paws. Two days afterward, the proliferation of CFSE^+^KJ1.26^+^ cells was analyzed by flow cytometry. **(A)** Representative staining results for KJ1.26^+^ effector T cells in the draining lymph nodes (DLNs) are graphed. **(B)** Representative CFSE dilution due to the proliferation of effector KJ1.26^+^ T cells was analyzed using FlowJo software (TreeStar, Ashland, OR, USA). Graph shows the same numbers of CD4^+^KJ1.26^+^ cells isolated from mice that received injections of saline (gray) or ova-Treg cells (bold). **(C)** Graphed numbers of KJ1.26^+^ proliferating cells in the DLNs. **(D)** CD90.1 congenic BALB/c mice received injections of CFSE-labeled, ova-specific effector CD4^+^ cells and 1 × 10^7^ ova-Treg cells (*n* = 8) or PBS (*n* = 5). The numbers of ova-Treg cells (CD90.2^+^) in the DLNs are shown. Differences were analyzed by Mann–Whitney *U* test with 95% confidence intervals.

We further investigated the *in vivo* suppressive mechanism of Tr1 cells, and notably their impact on the proliferation of effector T cells, using an ova-specific, delayed-type hypersensitivity (DTH) experimental model. The proliferation of antigen-specific effector T cells was monitored in the draining LN using the KJ1.26 antibody. As shown in Figure 5, injection of 1 × 10^6^ ova-Treg cells significantly dampened the proliferation of effector T cells into the DLNs, as revealed by the lower number of KJ1.26^+^ cells (Figure 5A) and the higher intensity of CFSE staining (Figure 5B). Indeed, we observed a significant decrease in the number of KJ1.26^+^ cells in the Tr1-treated mice compared with controls (Figure 5C).

This DTH experimental model was also used to monitor the homing properties of injected Tr1 cells. To this aim, a higher dose of ova-Treg cells (10 × 10^6^ per animal) was injected into CD90.1 congenic BALB/c mice, and injected ova-Treg cells were tracked using the CD90.2 marker. Ova-Treg cells (CD90.2^+^) were found in the mononuclear cell fractions of the blood and the spleen (data not shown), as well as in the DLNs (Figure 5D).

Altogether, these results suggest that one of the suppressive mechanisms used by ova-Treg cells *in vivo*, following their migration into the DLNs, is to dampen the proliferation of antigen-specific effector T cells.

## Discussion

Several lines of evidence indicate a link between Tr1 cells and clinical improvement in RA patients. First, clinical response to anti-TNF therapy has been associated with the differentiation of a potent population of iTreg cells with suppressive activity that is dependent on TGF-β and IL-10 [12]. Also, disease remission in RA patients is associated with restored numbers of peripheral blood plasmacytoid dendritic cells that are implicated in the maintenance of tolerance through the induction of IL-10–secreting Tr1 cells [39]. In the present study, we evaluated, for the first time to the best of our knowledge, the clinical benefit of adoptively transferred *in vitro* differentiated Tr1 cells specific for a locally expressed antigen in two preclinical models of arthritis.

A key question about the use of Treg cells in therapy is to determine whether antigen-specific or polyclonal Treg cells are more suppressive in various clinical settings. In order to induce suppression, Treg cells must first be activated via TCRs by recognition of specific antigen major histocompatibility complexes. Authors of several reports have demonstrated that high TCR diversity appears to be crucial for optimal Treg-cell capacity [40]. The improved suppressive potential is probably due to increased numbers of activated Treg cells, which is underscored by the facts that (1) wild-type CD4^+^CD25^+^ Treg cells have higher suppressive capacity in lymphopenic mice [7] and (2) relevant tissue-specific antigen must be present in the periphery in order to generate fully protective Treg cells [41]. Concerning antigen-specific Tr1 cells, as clearly demonstrated in an IBD experimental model, the antigen specificity of the Tr1 cells is required for their local activation to trigger their suppressive function, even if the antigen (ova) is not implicated in the inflammation of the colon. In our study, we chose a self-antigen specifically expressed in the joint—Col II—to trigger local activation of Tr1 cells.

First, we demonstrated the feasibility of producing mouse Col II–specific Tr1 cells using an experimental protocol previously used for the production of ova-specific Tr1 cells. The Tr1 cell identity of Col-Tregs was confirmed by their cytokine secretion profile (IL-10^high^IL-4^neg^IFN-γ^int^) and by their *in vitro* immunosuppressive activity, which was dependent on IL-10 and TGF-β production [20,26]. In contrast to thymus-derived CD4^+^CD25^+^ nTregs, Col-Treg cells upregulate FoxP3 and CD25 upon TCR activation and do not express CD62L, underlining their iTreg phenotype. However, as described for nTregs [42], activated Col-Treg cells do not express high levels of CD127. Overall, these mouse Col-Treg cells share similar characteristics with the human Col-Treg cells that we previously obtained from RA patients [22] and with ova-Treg cells derived from Crohn’s disease patients lymphocytes [21].

The data presented herein indicate that a single Col-Treg cell infusion leads to a significant decrease in clinical symptoms in both acute and chronic models of arthritis as measured by reductions in paw swelling, arthritic scores, clinical incidence and body weight loss, as well as by reduction of Col II–specific antibody titers. Interestingly, the results of a recent comparative study of antigen-specific nTregs and iTregs implicated greater efficacy for the latter, with a dose range similar to that which we used in our present study [43]. In both models used, the Col-Treg cells are detected in inflamed joints and axillary LNs following injection. These data provide evidence that Col-Treg cells are delivered to their site of action, (that is, in the arthritic joints), thus leading to a decrease in local inflammation. Our observations are similar to those of previous researchers who conducted distribution studies of infusion of CD4^+^CD25^+^ Treg cells in CIA, for which inhibition of local inflammation is associated with a decrease in IgG antibodies [6,7].

IL-10 has been described as the main mediator of Tr1 anti-inflammatory effects. Systemic and locally administered IL-10 have previously been reported to have clear therapeutic potency in CIA [44-50]. Interestingly, in those studies, the therapeutic effect of IL-10 on anti–Col II antibodies was associated with contradictory results that could be explained by the dose and timing of the IL-10 treatments. In our study, we found that the therapeutic effect of Col-Treg cells injected at the onset of clinical signs was associated with a global impact on anti–Col II IgG antibodies, whereas a preferential decrease in the anti–Col II IgG2a isotypes was monitored following earlier injection. IL-10 is also a potent suppressor of macrophage activation, and intraarticular IL-10 secretion has been reported to be associated with decreased proinflammatory cytokines [51]. The local IL-10–mediated decrease in TNF-α is a likely mechanism underlying the Tr1-protective effect observed in the CAIA experimental model, in which neutrophils and macrophages are important inflammatory cells and TNF-α and IL-1β secretion is pathogenic.

Extensive studies of the modes of action of Tr1 cells in the two experimental models fall outside the scope of our present study. However, to further investigate the *in vivo* suppressive function of the Tr1 cells, and notably their impact on the adaptive immune response, we provide evidence that ova-Treg cells significantly dampen the proliferation of antigen-specific CD4 effector T cells in DLNs. The antigen specificity of Tr1 cells is necessary to control their activation; however, we cannot exclude the possibility that the suppressive mechanism implies bystander suppression and impedes the proliferation of non-antigen-specific T effector cells. Although IL-10 has been considered to be the main mediator of a Tr1 anti-inflammatory effect for a long time, the concomitant expression of other anti-inflammatory cytokines, such IL-13, and immunomodulatory molecules, including GITR, CTLA-4, CD39 and granzyme B, suggest that Col-Treg cells display an arsenal of molecules able to suppress the autoimmune response through different modes of action, as previously described for nTregs and iTregs [33,52]. Indeed, in addition to suppression of effector T cells via IL-10 and TGF-β secretion, expression of PD-1 (programmed cell death 1 cell surface protein) and CTLA-4 [53], adenosine production by CD39^+^ Tr1 cells [54] and granzyme B–dependent killing of myeloid cells has previously been found to be associated with a suppressive function of human Tr1 cells. The latter cytolytic activity on macrophages and neutrophils, which are key inflammatory cells in the CAIA experimental model, is an additional mechanism underlying the therapeutic effect of Col-Tregs in the CAIA model.

The results of the present pharmacokinetic studies of injection of Col-Treg cells in arthritis confirm previous observations in which ova-Treg cells were used in an IBD model [26]. In severe combined immunodeficiency mice reconstituted with CD4^+^CD45RB^high^ T cells, the injected ova-Treg cells were detected in inflamed tissue after 4 weeks, but not later. In our present study, we demonstrate that Col-Treg cells were maintained after 1 week but barely detectable after 7 to 10 days. These data indicate that infusion of Col-Treg cells did not lead to uncontrolled expansion *in vivo*, even in the continuous presence of their specific antigens. This observation is crucial in the context of further cell therapy protocols using Col-Treg cells in RA patients because lymphoproliferation could be seen as a major potential pitfall of such treatment. Furthermore, we did not observe uncontrolled proliferation of injected cells in long-term experiments in which Col-Tregs were injected into naïve or arthritic mice, that is, under permanent antigen exposure in the absence or presence of inflamed tissue.

Because of the heterogeneity of human Treg cells, there is no consensus to date about which Treg population is optimally suited for their various clinical uses. One of the major obstacles to the implementation of clinical protocols using the various Tregs is the low frequency of Tregs in the peripheral blood, which necessitates *ex vivo* multiplication of the cells prior to their use *in vivo*. The most commonly used expansion protocol at present is based on polyclonal stimulation using anti-CD3/anti-CD28 beads in the presence of high doses of recombinant IL-2, which is supplemented in some protocols with rapamycin. However, the expansion is usually antigen-nonspecific. More appealing for clinical application is the concept of expanding antigen-specific Treg cells to control their activation. Importantly we previously demonstrated the feasibility of producing high numbers of clinical grade functional Col-Tregs in RA patients [22]. Altogether, our results strongly support a further evaluation of the therapeutic potential of Col-Treg cells in human disease. The use of Tr1 cells in human models of disease is enhanced by their multiple mechanisms of action, and their local immunosuppressive action, in contrast to the systemic and single-target approaches used in the development of large numbers of biologics for RA that are already approved or in development. Patients with severe RA that is refractory to biologics represent a high unmet medical need, and antigen-specific Tr1 cells could be an innovative approach to dampening inflammation and restoring immune tolerance to joint antigens in this patient population.

## Conclusions

The data presented herein demonstrate the therapeutic potential of antigen-specific Tr1 cells in two experimental models of arthritis and open great perspectives for the development of novel therapeutic approaches in the treatment of RA.

## Abbreviations

CAIA: Collagen antibody–induced arthritis; CFA: Complete Freund’s adjuvant; CIA: Collagen-induced arthritis; Col II: Collagen type II; Col-Treg: Collagen type II–specific type 1 regulatory T cell; i.p.: Intraperitoneal; i.v.: Intravenous; IBD: Inflammatory bowel disease; IFA: Incomplete Freund’s adjuvant; iTreg: Induced regulatory T cell; LN: Lymph node; nTreg: natural regulatory T cell; ova-Treg: Ovalbumin-specific type 1 regulatory T cell; RA: Rheumatoid arthritis; rhu: Recombinant human; Tr1: Type 1 regulatory T cell; Treg: Regulatory T cell.

## Competing interests

The authors declare that they have no competing interests.

## Authors’ contributions

AF and PLP are co-senior authors. All authors contributed to the study design, data acquisition and data analysis and interpretation. HA, DM, NB, CJ, AF, CJ and PLP contributed to the study concept and design, performed the statistical analysis, had full access to all of the data in the study and take responsibility for the integrity of the data and the accuracy of the data analysis. HA, DM, JQ, HB, MBJ, PBF, AMM, ALMB, SR and IM performed the *in vivo* experiments and analysis and interpretation of data. HA, DM, NB, CJ, AF and PLP contributed to the study concept and design, interpreted the data and wrote the manuscript. All authors read and approved the final manuscript.
